# Psychometric properties of the Beck Depression Inventory‐II in progressive supranuclear palsy

**DOI:** 10.1002/brb3.2344

**Published:** 2021-09-07

**Authors:** Sofia Cuoco, Arianna Cappiello, Filomena Abate, Maria Francesca Tepedino, Roberto Erro, Giampiero Volpe, Maria Teresa Pellecchia, Paolo Barone, Marina Picillo

**Affiliations:** ^1^ Neuroscience Section Department of Medicine Surgery and Dentistry, Center for Neurodegenerative Diseases (CEMAND) University of Salerno Fisciano Italy; ^2^ AOU S. Giovanni di Dio e Ruggi D'Aragona Neurology Unit Salerno Italy

**Keywords:** BDI‐II, depression, progressive supranuclear palsy, quality of life

## Abstract

**Objectives:**

Depression is one of the most common neuropsychiatric symptoms in progressive supranuclear palsy (PSP). Yet, few studies have examined the ability of available instruments to detect depressive symptoms in PSP. Aims of the present study were to (I) report psychometric properties of the Beck Depression Inventory Second Edition (BDI‐II) in PSP, (II) establish the BDI‐II cut‐off indicating the presence of depression in PSP and (III) describe clinical correlates as well as correlation with quality of life of depressive symptoms in PSP.

**Design, setting and participants:**

: At the Center for Neurodegenerative Diseases of the University of Salerno, Italy, the BDI‐II was validated in 62 PSP patients diagnosed according to the Movement Disorder Society criteria. Patients underwent a clinical interview, a motor evaluation, extensive cognitive and behavioral testing.

**Results:**

The mean BDI‐II total score was 15.92 ± 10.31. The internal consistency was high (Cronbach's alpha = 0.868); corrected item‐total correlation was >0.40 for the majority of items. The significant and moderate correlation of the BDI‐II with other tools evaluating depressive symptoms indicated adequate convergent validity of the scale. The satisfactory cut‐off to identify patients with clinically significant depression was >14.5. We also showed a correlation between higher scores on BDI‐II and lower quality of life, irrespective of motor and cognitive burden.

**Conclusion:**

In conclusion, the BDI‐II is a reliable and valid tool for the assessment of depression symptoms in PSP. Such data are useful to standardize studies of depression in PSP and to quantify the effectiveness of any interventions on this disabling symptom.

## Graphical abstract

We analyzed the psychometric properties of the second version of the Beck Depression Inventory Second Edition (BDI‐II) in a sample of 62 patients with progressive supranuclear palsy (PSP). The BDI‐II showed high internal consistency and good discriminant validity. The satisfactory cut‐off to identify patients with clinically significant depression was 14.5. Depressive symptoms in PSP had a significant impact on quality of life.

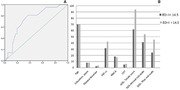

## 1 INTRODUCTION

Progressive supranuclear palsy (PSP) is a rapidly progressive neurodegenerative disease featured by postural instability and vertical gaze palsy as well as cognitive dysfunction and behavioral disturbances. New diagnostic criteria were recently released by the Movement Disorder Society (MDS) identifying different PSP phenotypes, as PSP with Richardson's syndrome (PSP‐RS), PSP with predominant parkinsonism (PSP‐P), PSP with predominant frontal presentation (PSP‐F), PSP with predominant progressive gait freezing (PSP‐PGF) and PSP with predominant speech/language disorder (PSP‐SL) (Bloise et al., 2014; Höglinger et al., 2017; Picillo, Cuoco, Tepedino, et al., 2019).

Depression is one of the most common neuropsychiatric symptoms in PSP. Irrespective of the different tool used, about 50% of PSP patients report depressive symptoms (APA, 2013; Esmonde et al., 1996; Gerstenecker et al., 2013; Menza et al., 1995; Schrag et al., 2010). Moreover, scant of data are available from the literature on the frequency of depression in PSP phenotypes. Specifically, as reported by Pellicano et al. (2017) and Assogna et al. (2019), patients with PSP‐RS did not differ to PSP‐P for depression symptoms, and depression was not a predictor of phenotypes.

However, although there are many rating scales that measure depressive symptoms in healthy individuals or neurological medical populations (Creighton et al., 2019; De Souza et al., 2010; Massai et al., 2018), disease‐tailored tools to detect depression in PSP are lacking and all studies that have so far investigated depression in PSP have used nonvalidated scales. Similarly, there is no guidance on any specific cut‐off to diagnose depression with currently available instruments.

Several specific disease features may hamper the evaluation of depressive symptoms in PSP such as motor and cognitive impairment and oculomotor difficulties. Thus, it would be advisable to verify the specific psychometric properties of the available tools to assess depression in this specific population of patients.

The Beck Depression Inventory Second Edition (BDI‐II) is one of the most common measures of depressive symptoms in neurodegenerative diseases (Wang & Gorenstein, 2012). It is a self‐assessment scale evaluating a relatively narrow range of symptoms also providing a rating of severity. To date, no data are available on the psychometric properties of the BDI‐II in PSP.

Aims of the present study were to (I) report psychometric properties of the BDI‐II in PSP, (II) establish a cut‐off for the BDI‐II indicating the presence of depression in PSP and (III) describe clinical correlates as well as correlation with quality of life of depressive symptoms in PSP.

## 2 METHODS

### 2.1 Patients

Between November 2015 and December 2019, consecutive cases of suspected PSP referred to the Center for Neurodegenerative Diseases (CEMAND) of the University of Salerno, Italy, were proposed a dedicated set of assessments including a clinical interview, a motor evaluation, extensive cognitive and behavioral testing (Picillo et al., 2018, 2020; Picillo, Cuoco, Tepedino et al., 2019; Picillo, Cuoco, Carotenuto, et al., 2019).

Patients were enrolled in the present study upon evaluation with the MDS criteria for PSP (Höglinger et al., 2017). As such, the MDS proposed diagnostic flowchart was applied by two specialists in movement disorders who also defined the PSP phenotype (26 PSP‐RS, 13 PSP‐P, 10 PSP‐corticobasalsyndrome [CBS], 7 PSP‐PGF and 4 PSP‐F) according to the predominant clinical features and expressed the degree of diagnostic certainty (Grimm et al., 2019; Höglinger et al., 2017; Picillo et al., 2018). Diagnosis as well as phenotypic attribution was verified for all patients during at least one sequent visit. All patients reached the diagnostic degree of probability but those—by definition—with PSP‐CBS. As PSP‐P, PSP‐CBS, PSP‐PGF and PSP‐F included a limited number of patients, those subtypes were grouped together as the other variant syndrome of PSP (vPSP = 36). Details on inclusion/exclusion criteria are available elsewhere (Picillo, Cuoco, Tepedino, et al., 2019). Specific additional inclusion criteria for the present study were (1) presence of an Italian‐speaking caregiver, (2) absence of severe significant language or speech impairment preventing even minimal communication with the examiner and (3) Mini‐Mental State Examination (MMSE) score >13.

The project was performed in accordance with the ethical standards laid down in the 1964 Declaration of Helsinki and was approved by the local Ethics Committee (Campania Sud) and each subject was included upon signature of the informed consent form.

### 2.2 Clinical and cognitive‐behavioral assessments

Severity of disease was rated with the progressive supranuclear palsy rating scale (PSP‐rs) including 28 items divided in six subscores (i.e., activities of daily living, behavior, bulbar, ocular, limb and gait/midline) (Golbe & Ohman‐Strickland, 2007).

Global cognitive functioning was evaluated with the Italian version of the Montreal Cognitive Assessment battery (MOCA) and the Clock drawing test (CDT) (Santangelo et al., 2015; Siciliano et al., 2016).

An independent neuropsychologist expert in movement disorders blinded to the rating scales attributed the diagnosis of depression to each patient based on a clinical structured interview following the diagnostic criteria of Diagnostic and Statistical Manual of Mental Disorders, 5th Edition (DSM‐5). Of note, we did not consider the exclusion criteria “presence of other organic pathologies” proposed by the DSM‐5 (APA, 2013).

Depressive symptoms were evaluated with the BDI‐II, and the item 4 of Natural History and Neuroprotection in Parkinson Plus Syndromes Parkinson Plus Scale (NNIPPS) (Payan et al., 2011).

The BDI‐II consists of 21 questions exploring depressive symptoms in the previous two weeks; all items are scored on 4‐point Likert Scale ranging from 0 to 3. The total score ranges from 0 to 63 (with higher scores indicating more severe depression). In healthy subjects, the cut‐off for the presence of depression has been set at 11. Two subscores can be computed: the affective somatic including items 4, 10, 11, 12, 13, 15, 16, 17, 18, 19, 20, 21 and the cognitive one including the remaining items (Beck et al., 1996). Of note, PSP patients were actively supported in the compilation of the BDI‐II in order to reduce the impact of ocular, motor and cognitive difficulties.

Furthermore, quality of life was evaluated with the Progressive Supranuclear Palsy Quality of Life Questionnaire (PSP‐QoL) (Picillo, Cuoco, Amboni, Bonifacio, Bruschi, et al., 2019) and apathy with Apathy Evaluation Scale (AES).

Patients were actively supported in the fulfillment of all self‐assessments in order to reduce the impact of ocular, motor and cognitive difficulties. Examples of active support for scales included reading the items and responses, deepening for ambiguous responses and exploring the overlap of motor and emotional‐behavioral symptoms. Also, additional information was gathered from the caregiver.

### 2.3 Statistical analysis

Demographic, clinical and cognitive variables were compared between groups with the Mann–Whitney or chi‐square tests as appropriate.

Quality of data obtained with the questionnaire BDI‐II was evaluated by computing percentage of missing or invalid items; a percentage of <5% of missing values was considered as an index of acceptable data quality. Moreover, data quality was assessed using mean, median, skewness (criterion: −1 to +1) and extent of ceiling and floor effects. Floor and ceiling effects <15% were defined as optimal.

Internal consistency of the BDI‐II and its subscores were evaluated by Cronbach's alpha; a value ≥0.70 was considered as acceptable. Scaling assumptions referring to the correct grouping of items and the appropriateness of their summed score were checked using corrected item‐total correlation (standard ≥0.40).

Precision was evaluated by computing the standard error of measurement (SEM = SD √[1 – Cronbach's alpha]); a SEM value ≤1/2 standard deviation was taken as the criterion of acceptable precision.

Nonparametric Spearman's correlation was used to measure construct validity of the BDI‐II total score (internal validity: correlation with BDI‐II subscores; convergent validity: with item 4 of the NNIPPS; discriminant validity: with MOCA and CDT). Effect size of the correlation coefficients was defined with the following criteria: Spearman's r < 0.3 weak; Spearman's r = 0.3−0.5 moderate; Spearman's r > 0.5 strong. We analyzed the mean responses to individual items and the distribution of symptom severity in order to study the items with the most frequent high responses.

Diagnostic accuracy of the BDI‐II was evaluated with the receiver operating characteristics (ROC) analysis. Here, the clinical diagnosis of depression according to the DSM‐5 criteria was used as gold standard in the estimation of sensitivity, specificity and for determining the optimal cut‐off score. Furthermore, also criterion validity of BDI‐II was explored with reference to clinical diagnostic criteria for depression. For this purpose, we used Mann–Whitney test to compare BDI‐II scores between patients with and without depression according to DSM‐5 criteria.

Finally, clinical correlates as well as correlation with quality of life of depressive symptoms in PSP were explored. Demographic and clinical features were compared between patients with (D+) and without depression (D‐) as determined with the newly identified cut‐off of the BDI‐II for PSP. Then, the correlation between depression and demographic, motor and cognitive variables and quality of life was investigated with multiple logistic regression analysis (dependent variable: D+ status; independent variables: age and gender, PSP‐rs, PSP phenotype [PSP‐RS vs. vPSP], MOCA and PSP‐QoL total score).

All analyses were performed using SPSS version 20, (SPSS Inc., Chicago, IL, USA) with *p*‐value < .05 considered statistically significant.

## 3 RESULTS

Eighty‐three patients were screened for the present study and twenty‐one were excluded based on enrollment criteria, that is, 14 for unconfirmed diagnoses of PSP, six for MMSE < 13, one qualifying for PSP‐SL for the presence of significant language impairment. Thus, sixty‐two PSP patients (33 men) were included and their demographic and clinical features are shown in Table 1.

**TABLE 1:** Demographic and clinical features of the enrolled cohort

	Whole sample *N* = 62 (M = 33) Median (IQR)
Age, years	70 (10)
Education, years	8 (8)
Disease duration, years	3 (3)
PSP‐rs	36 (18)
MOCA	18 (8)
CDT	5 (4)
PSP‐QoL total score	82 (52)
PSP‐QoL Mental subscale	45 (33)
PSP‐QoL Physical subscale	34 (34)

Abbreviations: CDT, Clock drawing test; IQR, interquartile range; M, male; MOCA, Montreal Cognitive Assessment; N, number; PSP‐QoL, progressive supranuclear palsy quality of life questionnaire; PSP‐rs, progressive supranuclear palsy rating scale.

### 3.1 Psychometric properties of BD‐II in PSP

All data collected with the BDI‐II were computable and there were no missing values. The mean (±standard deviation) BDI‐II score was 15.92 ± 10.31 and the median (interquartile range) was 14 (15) (difference between mean score and the median was 1.92).

One hundred percent of data were totally computable and there were no missing values (0%). In the whole PSP sample, neither the ceiling nor the floor effects were observed for the BDI‐II total score (lowest possible score = 0, 1.6%; highest possible score = 43, 1.6%). The skewness of BDI‐II total score was 0.650 (criterion: −1 to +1).

Cronbach's alpha for the BDI‐II total score was 0.868 and thus, it was considered acceptable for internal consistency. No improvement in Cronbach's alpha was observed upon removal of any item. Item‐total correlation was ≥0.40 for all items except for questions 6, 8, 16 and 21. Also, item 3 of BDI‐II did not show any significant correlation with total score (see Table S1). Cronbach's alpha for both somatic‐affective and cognitive subscores was considered acceptable for internal consistency (0.808 and 0.737, respectively).

Internal validity was confirmed by the significant correlation of the BDI‐II total score with both BDI‐II cognitive subscore and somatic‐affective subscore. Both subscores correlated with each other (*p* ≤ .001) (see Table S1).

The standard error of measurement (SEM) value for BDI‐II total score was 3.82 (SEM = SD √[1 – Cronbach's alpha]).

The BDI‐II total score showed convergent validity with the item 4 of the NNIPPS (Table 2). Divergent validity was demonstrated by the lack of correlation between the total BDI‐II score with the MOCA and CDT (Table 2). There was a significant correlation between BDI‐II and AES (rho = 0.602, *p *< .001).

**TABLE 2:** Convergent and divergent validity of the BDI‐II total score

	BDI‐IITotal score(Spearman's *r*)	*p*‐Value
Age	0.012	.927
Education, years	0.015	.909
Disease duration	0.197	.138
PSP‐rs	0.419	**.001**
MOCA	−0.244	.067
CDT	−0.116	.394
NNIPPS‐item 4 depression	0.343	**.009**
PSP‐QoL total score	0.555	**.000**

*Note*: Statistically significant differences are indicated in bold.

Abbreviations: NNIPPS‐item 4, Natural History and Neuroprotection in Parkinson Plus Syndromes Parkinson Plus Scale, item 4 for depression symptoms; CDT, Clock drawing test; MOCA,, Montreal Cognitive Assessment; PSP‐rs, progressive supranuclear palsy rating scale; PSP‐QoL, progressive supranuclear palsy quality of life.

**FIGURE 1:**
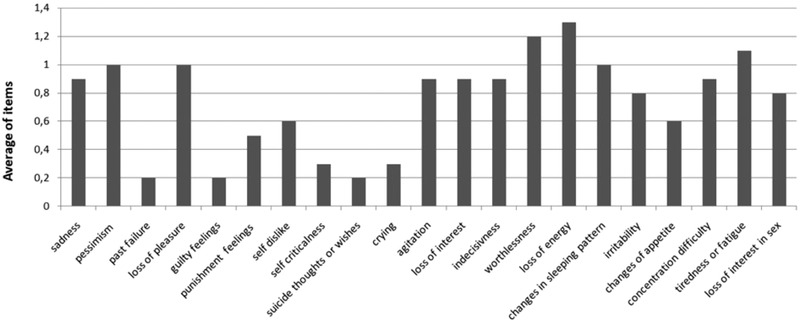
The average of the responses to the single items of Beck Depression Inventory Second Edition (BDI‐II)

The average score for each single item is shown in Figure 1. The lowest scores were given to the items investigating past failure (0.17 ± 4.0), guilt feelings (0.21 ± 5.0) and suicidal thoughts or wishes (0.25 ± 6.0). The highest responses were given to the items investigating loss of energy (1.29 ± 1.0), worthlessness (1.19 ± 1.1), tiredness or fatigue (1.09 ± 1.0), pessimism (0.95 ± 9.0), loss of pleasure (0.98 ± 1.0) and changes in sleeping pattern (1.04±1.0). The higher frequency of high responses (score 3) was in the items investigating the loss of energy (16.1%), worthlessness (21%) and loss of interest in sex (24.2%) (see Table S2).

Although half of PSP‐RS scored higher than 14.5, prevalence of depression was not different according to PSP phenotypes (*p* = .388) (Figure S1).

### 3.2 Establish the BDI‐II cut‐off indicating the presence of depression in PSP

According to DSM‐5 criteria, 21 patients were diagnosed as affected by depression (34%). Patients with depression diagnosed with DSM‐5 showed higher PSP‐rs scores but similar demographic and cognitive features as compared with patients without depression (*p* > .05) (Table 3).

**TABLE 3:** Demographic and clinical features of patients with and without depression by DSM‐5

	Patients with depressionmedian (IQR)	Patients without depressionmedian (IQR)	*p*‐Value
Age	67.00 (17)	68.00 (10)	.300
Education, years	11.50 (9)	8.00 (8)	.391
Disease duration	4.00 (5)	3.00 (3)	.515
PSP‐rs	44.00 (37)	35.00 (16)	**.011**
MOCA	17.00 (7)	17.00 (9)	.256
CDT	5.00 (4)	5.00 (5)	.281

*Notes*: Data are in median (Interquartile range = IQR), unless otherwise specified. Statistically significant differences are indicated in bold.

Abbreviations: CDT, Clock drawing test; DSM‐5: Statistical Diagnostic Manual of Psychiatry – 5th Edition; MOCA, Montreal Cognitive Assessment; PSP‐rs, progressive supranuclear palsy rating scale.

**FIGURE 2 (a):**
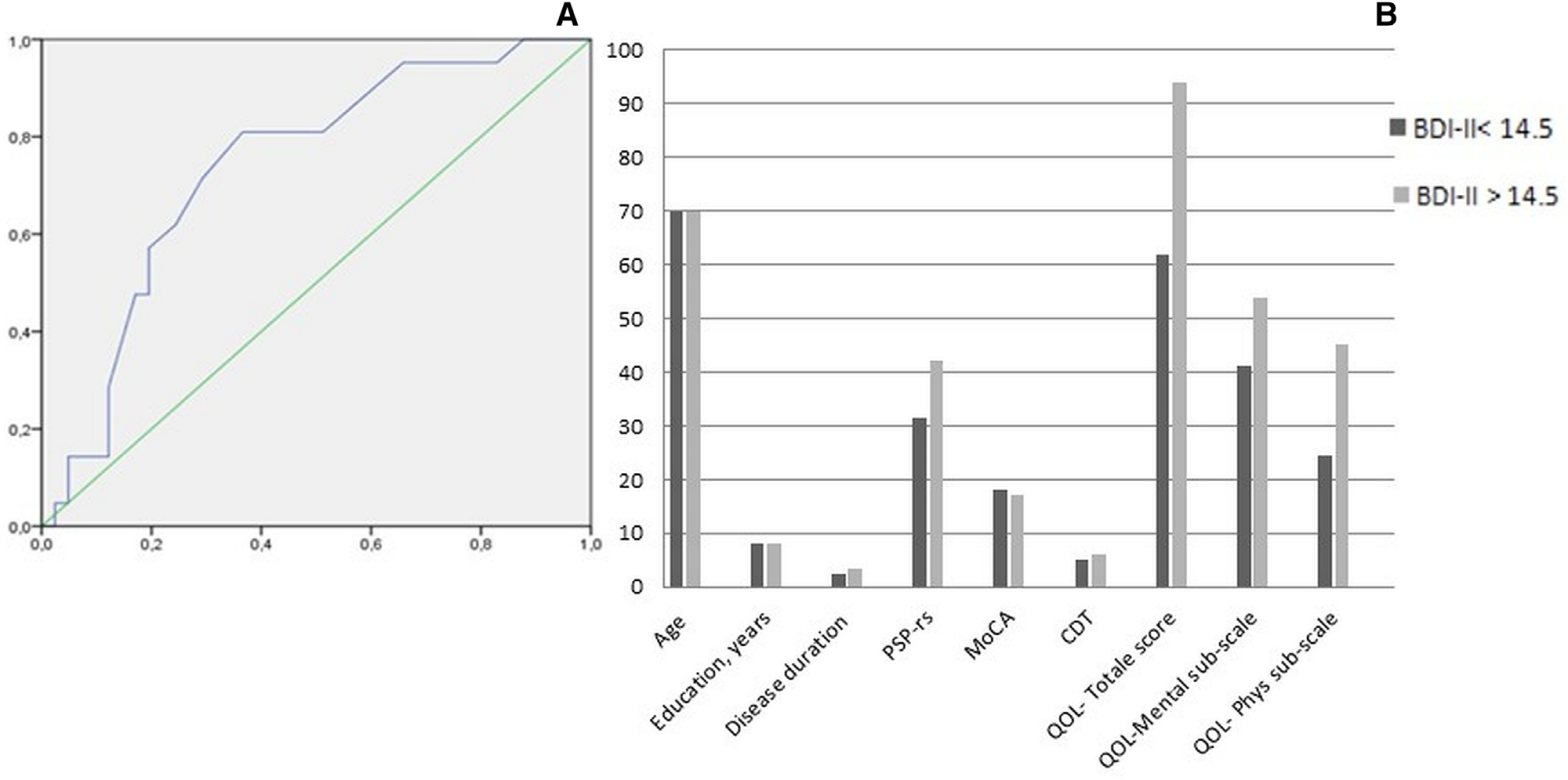
Results from receiver operating characteristics (ROC) analysis for the ability of the Beck Depression Inventory Second Edition (BDI‐II) cut‐off of 14.5 to detect clinically significant depression. y axis: sensitivity; x axis: specificity. (b) Comparison of demographic and clinical features between depressed (D+) and not depressed (D–). *p < .05. Abbreviations: CDT, Clock drawing test; D+, BDI‐II > 14.5; D–, BDI‐II < 14.5; MOCA, Montreal Cognitive Assessment; PSP‐QoL, progressive supranuclear palsy quality of life questionnaire; PSP‐rs, progressive supranuclear palsy rating scale

ROC analysis was used to assess the discriminatory power of the BDI‐II in identifying clinical significant depression. The determined optimal cut‐off was 14.5, showing 71% sensitivity, 71% specificity, 56% positive predictive value (PPV), 83% negative predictive value (NPV) and 71% diagnostic accuracy (Figure 2a). This cut‐off determined an area under the curve (AUC) with a discriminatory power of 73.9% (95% confidence interval [CI], 61.1%–86.7%).

Supporting criterion validity, PSP patients affected by depression according to DSM‐5 presented higher BDI‐II total scores (22 [13] vs. 12 [11], *p* = .002).

### 3.3 Clinical correlates and correlation with quality of life of depression in PSP

According to the newly identified cut‐off (14.5), patients were divided in depressed (D+) and not depressed (D‐). D+ reported higher scores on PSP‐QoL total score (94.0 [29.5] vs. 62.0 (31.50), *p* = .002), physical (45.0 [17.5] vs. 24.5 [20.50], *p* < .001) and mental (54.0 [33.75] vs. 41.0 [20.5], *p* = .012) subscales and PSP‐rs total score (42.0 [19] vs. 31.5 [19], *p* = .017) (Figure 2b). Distribution of disease phenotype (PSP‐RS and vPSP) was not different between the two groups (PSP‐RS: 48.1% in D+ and 37.1% in D‐, *p* = .383).

Multiple logistic regression showed that, among the different independent variables analyzed, only the PSP‐QoL total score was a significant predictor of D+ status (β = 0.035, standard error [SE] = 0.017, *p* = .042).

## 4 DISCUSSION

Herein, we measured the acceptability, validity and reliability of the BDI‐II in assessing depressive symptoms in PSP.

However, as reported in other neurodegenerative diseases (Creighton et al., 2019; De Souza et al., 2010; Massai et al., 2018), it is useful to have scales validated on a specific clinic sample in order to avoid unsuitable items and to reduce the influence induced by the overlap between motor and nonmotor symptoms. The use of a reliable and valid tool is essential in clinical practice and in the measurement of specific outcomes (Küçükdeveci et al., 2011; Streiner & Norman, 2008). In fact, several questionnaires are available to measure depression in patients with neurodegenerative diseases, but the literature shows that the same instrument does not always have the same validity in different groups of patients. For example, the validity analysis of the Geriatric Depression Scale (GDS‐30) showed good properties for Parkinson Disease (PD) (Massai et al., 2018) but less validity for patients with Alzheimer Disease (AD) (Debruyne et al., 2009).

The BDI‐II as a whole showed high acceptability since data were computable for 100% and the percentage of missing values was 0% for all items. The adequate acceptability is also supported by the absence of both ceiling and floor effects for the BDI‐II total score. Several issues should be taken into account when evaluating acceptability of instruments assessing depression in PSP. First, hypokinesia and psychomotor slowing may have an impact on the results (Santamaria et al., 1986). Furthermore, oculomotor and cognitive impairment may interfere with the self‐compilation of questionnaires (Santangelo et al., 2018). Finally, some of the components of the BDI‐II as sleep changes and lack of energy overlap with PSP symptoms. Hence, the active support provided by an expert neuropsychologist in filling out the scale as well as the caregiver input were both pivotal in determining such high acceptability rate.

The internal consistency of BDI‐II is high and acceptable (Cronbach's alpha = 0.868; item BDI‐II total score correlation ≥0.40 for the majority of items). Moreover, the internal consistency of cognitive and somatic‐affective factors is high and acceptable (Cronbach's alpha = 0.737 and 0.808, respectively) and close to values obtained in a previous study using the BDI‐I in patients with Parkinson's disease (Visser et al., 2006). The lack of significant item‐total correlation for few items may suggest that either such questions were not able to adequately measure the related problems or the corresponding issues were less pronounced compared with others included in the questionnaire. Complementary, some of these items (i.e., 16 and 21) presented an adequate interrelation with the somatic‐affective factor of BDI‐II, thus suggesting a greater correlation with the corresponding subscore than with the total score.

The degree of precision of measurement, expressed in terms of SEM, is adequate suggesting the BDI‐II values in our sample are an accurate estimation of the BDI values in the PSP population.

As for convergent and divergent construct validity, the BDI‐II total score showed insignificant association with demographic variables, suggesting that the scale is suitable for PSP patients of any age, education and disease duration. Notwithstanding, since patients with MMSE <13 were excluded from the present study, the BDI‐II should not be administered to detect depressive symptoms in patients with significant cognitive impairment. The adequate construct validity of the BDI‐II was supported by a moderate correlation with the item 4 of the NNIPPS.

On the other hand, we found that depression correlated with apathy. Depression and apathy are the most frequent neuropsychiatric disorders in neurodegenerative diseases and their co‐occurrence is expected in many parkinsonian syndromes (Camargo et al., 2018; Santangelo et al., 2018) and dementias (Collins et al., 2020). Distinguishing apathy from depression can be a major challenge due to an overlap in symptom, despite many studies suggested that they are separate entities underlined by different neural networks (Isella et al., 2002; Kirsch‐Darrow et al., 2006; Mayberg, 1994; Pluck & Brown, 2002). Further studies are needed to validate specific scales measuring apathy in PSP to have uniform and reliable results. As for diagnostic accuracy, our data indicate that the cut‐off of 14.5 is helpful in identifying patients with clinical significant depression. A similar cut‐off has been proposed for Parkinson's disease (Visser et al., 2006). However, we are also aware that the BDI‐II is a screening scale and, as such, is supposed to accompany clinical evaluation and not to be used as a stand‐alone.

However, although there are many rating scales that measure depressive symptoms in healthy individuals or neurological medical populations (Creighton et al., 2019; De Souza et al., 2010; Massai et al., 2018), disease‐tailored tools to detect depression in PSP are lacking and all studies that have so far investigated depression in PSP have used nonvalidated scales.

Finally, we performed a comprehensive evaluation of the clinical correlates of depressive status in PSP. In line with previous data (Schrag et al., 2010; Winter et al., 2011), we found a significant correlation between depressive status and quality of life as evaluated with the PSP‐QoL. The novelty of the present study consists in the preliminary evaluation of the psychometric properties of the tool used to measure depression in PSP. Similarly, the PSP‐QoL underwent language validation in an independent set of Italian patients (Picillo Cuoco, Amboni, Bonifacio, Bruschi, et al., 2019). Indeed, such formal validation of all the scales used represents a point of strength of the present work. As for the correlation with motor disability, although the BDI‐II total score presented a moderate convergent validity with disease severity assessed with PSP‐rs, regression analysis did not confirm association between depressive status and PSP‐rs. Furthermore, in line with previous data, we failed to find significant differences in depressive symptoms among PSP phenotypes (Picillo, Cuoco, Tepedino, et al., 2019).

Taken together our data would suggest the BDI cut‐off of 14.5 can be helpful in identifying a subgroup of patients presenting clinically significant depressive symptoms irrespective of motor and cognitive burden. In such scenarios, we argue that this subgroup of patients may present specific dysfunction in the mesolimbic dopaminergic pathways and/or fronto‐striatal connections similarly to patients affected by other movement disorders showing depressive symptoms (Cummings, 1993; Winter et al., 2011).

Depression has been identified as a possible predictor of shorter survival in PSP (Bloise et al., 2014; dell’ Aquila et al., 2013; Gerstenecker et al., 2013). As such, a better characterization of depression can potentially improve the clinical care of these patients. Also, as neuropsychiatric symptoms have a detrimental effect also on caregivers’ quality of life (Picillo, uoco, Amboni, Bonifacio, Bruno, et al., 2019; Uttl et al., 1998), increasing awareness of the behavioral burden in PSP can improve the quality of life of both patients and caregivers. However, we acknowledge as a limitation of the present work the exclusion of patients presenting with significant speech/language disorder.

## 5 CONCLUSION

Depressive symptoms are intrinsic to movement disorders. Our data demonstrate that the BDI‐II is an accurate and reliable tool to measure depressive symptoms in PSP, and a cut‐off of 14.5 can help in identifying a clinically significant depressive status irrespective of motor and cognitive burden. Such data are useful to standardize studies of depression in PSP and to quantify the effectiveness of any interventions. We also confirm the correlation between depression and quality of life in PSP. Neuroimaging studies are needed to confirm depressive symptoms in PSP are underlined by specific network dysfunctions.

## AUTHOR CONTRIBUTIONS

Substantial contributions to the conception or design of the work, analysis and interpretation of data for the work and drafting the work: Sofia Cuoco. Acquisition of data for the work: Arianna Cappiello, Filomena Abate, Maria Francesca Tepedino and Giampiero Volpe. Revising the data critically for important intellectual content: Roberto Erro, Maria Teresa Pellecchia and Paolo Barone. Substantial contributions to the conception or design of the work, analysis and interpretation of data for the work, revising the data critically for important intellectual content, and final approval of the version to be published: Marina Picillo.

## CONFLICT OF INTEREST

The authors declare no conflict of interest. Marina Picillo is supported by the Michael J Fox Foundation for Parkinson's research; Prof Paolo Barone received consultancies as a member of the advisory board for Zambon, Lundbeck, UCB, Chiesi, Abbvie and Acorda; Dr Roberto Erro received consultancies from Zambon and honoraria from TEVA.

### PEER REVIEW

The peer review history for this article is available at https://publons.com/publon/10.1002/brb3.2344

## Supporting information

Figure S1

Table S1

Table S2

## Data Availability

The data that support the findings of this study are available on request from the corresponding author. The data are not publicly available due to privacy or ethical restrictions.

## References

[brb32344-bib-0001] APA, American Psychiatric Association (2013). Diagnostic and statistical manual of mental disorders (5th ed.) (DSM‐5). American Psychiatric Association.

[brb32344-bib-0002] Assogna, F. , Pellicano, C. , Cravello, L. , Savini, C. , Macchiusi, L. , Pierantozzi, M. , Stefani, A. , Mercuri, B. , Caltagirone, C. , Pontieri, F. E. , & Spalletta, G. (2019) Alexithymia and anhedonia in early Richardson's syndrome and progressive supranuclear palsy with predominant parkinsonism. Brain and Behavior, 9(12), e01448. 10.1002/brb3.1448. 31743601 PMC6908877

[brb32344-bib-0003] Beck, A. T. , Steer, R. A. , & Brown, G. K. (1996) Beck depression inventory (2nd ed.). Psychological Corporation.

[brb32344-bib-0004] Bloise, M. C. , Berardelli, I. , Roselli, V. , Pasquini, M. , Stirpe, P. , Colosimo, C. , Berardelli, A. , & Fabbrini, G. . (2014). Psychiatric disturbances in patients with progressive supranuclear palsy: A case‐control study. Parkinsonism & Related Disorders, 20(9), 965–968. 10.1016/j.parkreldis.2014.05.015 24954060

[brb32344-bib-0005] Camargo, C. H. F. , Serpa, R. A. , Jobbins, V. A. , Berbetz, F. A. , & Sabatini, J. S. (2018). Differentiating between apathy and depression in patients with parkinson disease dementia. American Journal of Alzheimer's Disease and Other Dementias, 33(1), 30–34. 10.1177/1533317517728333 PMC10852426 28871794

[brb32344-bib-0006] Collins, J. D. , Henley, S. M. D. , & Suárez‐González, A. (2020). A systematic review of the prevalence of depression, anxiety, and apathy in frontotemporal dementia, atypical and young‐onset Alzheimer's disease, and inherited dementia. International Psychogeriatrics, 1–20. 10.1017/S1041610220001118 32684177

[brb32344-bib-0007] Creighton, A. S. , Davison, T. E. , & Kissane, D. W. (2019). The psychometric properties, sensitivity and specificity of the geriatric anxiety inventory, hospital anxiety and depression scale, and rating anxiety in dementia scale in aged care residents. Aging & Mental Health, 23(5), 633–642 29470096 10.1080/13607863.2018.1439882

[brb32344-bib-0008] Cummings, J. L. (1993). Frontal‐subcortical circuits and human behavior. Archives of Neurology, 50(8), 873–880. 10.1001/archneur.1993.00540080076020 8352676

[brb32344-bib-0009] De Souza, J. , Jones, L. A. , & Rickards, H. (2010). Validation of self‐report depression rating scales in Huntington's disease. Movement Disorders: Official Journal of the Movement Disorder Society, 25(1), 91–96. 10.1002/mds.22837 19908314

[brb32344-bib-0010] Debruyne, H. , Van Buggenhout, M. , Le Bastard, N. , Aries, M. , Audenaert, K. , De Deyn, P. P. , & Engelborghs, S. (2009) Is the geriatric depression scale a reliable screening tool for depressive symptoms in elderly patients with cognitive impairment? International Journal of Geriatric Psychiatry, 24(6), 556–562. 10.1002/gps.2154 19132643

[brb32344-bib-0011] Dell'Aquila, C. , Zoccolella, S. , Cardinali, V. , de Mari, M. , Iliceto, G. , Tartaglione, B. , Lamberti, P. , & Logroscino G. (2013). Predictors of survival in a series of clinically diagnosed progressive supranuclear palsy patients. Parkinsonism & Related Disorders, 19(11), 980–985. 10.1016/j.parkreldis.2013.06.014 23968651

[brb32344-bib-0012] Esmonde, T. , Giles, E. , Gibson, M. , & Hodges, J. R. (1996). Neuropsychological performance, disease severity, and depression in progressive supranuclear palsy. Journal of Neurology, 243(9), 638–643. 10.1007/BF00878659 8892064

[brb32344-bib-0013] Gerstenecker, A. , Duff, K. , Mast, B. , Litvan, I., & ENGENE‐PSP Study Group (2013). Behavioral abnormalities in progressive supranuclear palsy. Psychiatry Research, 210(3), 1205–1210. 10.1016/j.psychres.2013.08.045 24035530 PMC3840159

[brb32344-bib-0014] Golbe, L. I. , & Ohman‐Strickland, P. A. (2007). A clinical rating scale for progressive supranuclear palsy. Brain: A Journal of Neurology, 130(Pt 6), 1552–1565. 10.1093/brain/awm032 17405767

[brb32344-bib-0015] Grimm, M.‐J. , Respondek, G. , Stamelou, M. , Arzberger, T. , Ferguson, L. , Gelpi, E. , Giese, A. , Grossman, M. , Irwin, D. J. , Pantelyat, A. , Rajput, A. , Roeber, S. , Swieten, J. C. , Troakes, C. , Antonini, A. , Bhatia, K. P. , Colosimo, C. , Eimeren, T. , Kassubek, J. , … Höglinger, G. U. (2019). How to apply the movement disorder society criteria for diagnosis of progressive supranuclear palsy. Movement Disorders: Official Journal of the Movement Disorder Society, 34(8), 1228–1232. 10.1002/mds.27666 30884545 PMC6699888

[brb32344-bib-0016] Höglinger, G. U. , Respondek, G. , Stamelou, M. , Kurz, C. , Josephs, K. A. , Lang, A. E. , Mollenhauer, B. , Müller, U. , Nilsson, C. , Whitwell, J. L. , Arzberger, T. , Englund, E. , Gelpi, E. , Giese, A. , Irwin, D. J. , Meissner, W. G. , Pantelyat, A. , Rajput, A. , Van Swieten, J. C. , … Litvan, I. (2017). Clinical diagnosis of progressive supranuclear palsy: The movement disorder society criteria. Movement Disorders: Official Journal of the Movement Disorder Society, 32(6), 853–864. 10.1002/mds.26987 28467028 PMC5516529

[brb32344-bib-0017] Isella, V. , Melzi, P. , Grimaldi, M. , Iurlaro, S. , Piolti, R. , Ferrarese, C. , Frattola, L. , & Appollonio, I. (2002) Clinical, neuropsychological, and morphometric correlates of apathy in Parkinson's disease. Movement Disorders, 17(2), 366–371. 10.1002/mds.10041 11921125

[brb32344-bib-0018] Kirsch‐Darrow, L. , Fernandez, H. H. , Marsiske, M. , Okun, M. S. , & Bowers, D. (2006) Dissociating apathy and depression in Parkinson disease. Neurology, 67(1), 33–38. 10.1212/01.wnl. 16832074 PMC2911155

[brb32344-bib-0019] Küçükdeveci, A. , Tennant, A. , Grimby, G. , & Franchignoni, F. (2011) Strategies for the evaluation and measurement of outcomes in physical and rehabilitative medicine: A review educational. Journal of Rehabilitation Medicine, 43(8), 661–672. 10.2340/16501977-0844 21687922

[brb32344-bib-0020] Massai, P. , Colalelli, F. , Sansoni, J. , Valente, D. , Tofani, M. , Fabbrini, G. , Fabbrini, A. , Scuccimarri, M. , & Galeoto, G. (2018). Reliability and validity of the geriatric depression scale in Italian subjects with parkinson's disease. Parkinson's Disease, 2018, 7347859; 10.1155/2018/7347859 PMC6093010 30155239

[brb32344-bib-0021] Mayberg, H. S. (1994) Frontal lobe dysfunction in secondary depression. The Journal of Neuropsychiatry and Clinical Neurosciences, 6(4), 428–442. 7841814 10.1176/jnp.6.4.428

[brb32344-bib-0022] Menza, M. A. , Cocchiola, J. , & Golbe, L. I. (1995). Psychiatric symptoms in progressive supranuclear palsy. Psychosomatics, 36(6), 550–554. 10.1016/S0033-3182(95)71610-3 7501785

[brb32344-bib-0023] Payan, C. A. M. , Viallet, F. , Landwehrmeyer, B. G. , Bonnet, A. M. , Borg, M. , Durif, F. , Lacomblez, L. , Bloch, F. , Verny, M. , Fermanian, J. , Agid, Y. , Ludolph, A. C. , Leigh, P. N. , & Bensimon, G. (2011). Disease severity and progression in progressive supranuclear palsy and multiple system atrophy: Validation of the NNIPPS–Parkinson Plus Scale. Plos One, 6(8), e22293. 10.1371/journal.pone.0022293 21829612 PMC3150329

[brb32344-bib-0024] Pellicano, C. , Assogna, F. , Cellupica, N. , Piras, F. , Pierantozzi, M. , Stefani, A. , Cerroni, R. , Mercuri, B. , Caltagirone, C. , Pontieri, F. E. , & Spalletta, G. , (2017) Neuropsychiatric and cognitive profile of early Richardson's syndrome, Progressive Supranuclear Palsy‐parkinsonism and Parkinson's disease. Parkinsonism & Related Disorders, 45, 50–56. 10.1016/j.parkreldis.2017.10.002 29037499

[brb32344-bib-0025] Picillo, M. , Erro, R. , Cuoco, S. , Tepedino, M. F. , Manara, R. , Pellecchia, M. T. , & Barone, P. (2018). MDS PSP criteria in real‐life clinical setting: Motor and cognitive characterization of subtypes. Movement Disorders: Official Journal of the Movement Disorder Society, 33(8), 1361–1365. 10.1002/mds.27408 29984518

[brb32344-bib-0026] Picillo, M. , Cuoco, S. , Tepedino, M. F. , Cappiello, A. , Volpe, G. , Erro, R. , Santangelo, G. , Pellecchia, M. T. , & Barone, P. (2019). Motor, cognitive and behavioral differences in MDS PSP phenotypes. Journal of Neurology, 266(7), 1727–1735. 10.1007/s00415-019-09324-x 30989369

[brb32344-bib-0027] Pluck, G. C. (2002) Apathy in Parkinson's disease. Journal of Neurology, Neurosurgery, and Psychiatry, 73(6), 636–642. 10.1136/jnnp.73.6.636 PMC1757348 12438462

[brb32344-bib-0028] Picillo, M. , Cuoco, S. , Carotenuto, I. , Abate, F. , Erro, R. , Volpe, G. , Pellecchia, M. T. , Catricalà, E. , Cappa, S. , & Barone, P. (2019). Clinical use of SAND battery to evaluate language in patients with Progressive Supranuclear Palsy. Plos One, 14(10), e0223621. 10.1371/journal.pone.0223621 31603934 PMC6788681

[brb32344-bib-0029] Picillo, M. , Cuoco, S. , Amboni, M. , Bonifacio, F. P. , Bruschi, F. , Carotenuto, I. , De Micco, R. , De Rosa, A. , Del Prete, E. , Di Biasio, F. , Elifani, F. , Erro, R. , Fabbri, M. , Falla, M. , Franco, G. , Frosini, D. , Galantucci, S. , Lazzeri, G. , Magistrelli, L. , … Barone, P. (2019). Validation of the Italian version of the PSP quality of life questionnaire. Neurological Sciences: Official Journal of the Italian Neurological Society and of the Italian Society of Clinical Neurophysiology, 40(12), 2587–2594. 10.1007/s10072-019-04010-2 31350659

[brb32344-bib-0030] Picillo, M. , Cuoco, S. , Amboni, M. , Bonifacio, F. P. , Bruno, A. , Bruschi, F. , Cappiello, A. , De Micco, R. , De Rosa, A. , Di Biasio, F. , Elifani, F. , Erro, R. , Fabbri, M. , Falla, M. , Franco, G. , Frosini, D. , Galantucci, S. , Lazzeri, G. , Magistrelli, L. , … Barone, P. (2019). Validation of the Italian version of carers' quality‐of‐life questionnaire for parkinsonism (PQoL Carer) in progressive supranuclear palsy. Neurological Sciences: Official Journal of the Italian Neurological Society and of the Italian Society of Clinical Neurophysiology, 40(10), 2163–2169. 10.1007/s10072-019-03944-x 31190253

[brb32344-bib-0031] Picillo, M. , Tepedino, M. F. , Abate, F. , Erro, R. , Ponticorvo, S. , Tartaglione, S. , Volpe, G. , Frosini, D. , Cecchi, P. , Cosottini, M. , Ceravolo, R. , Esposito, F. , Pellecchia, M. T. , Barone, P. , & Manara, R. (2020). Midbrain MRI assessments in progressive supranuclear palsy subtypes. Journal of Neurology, Neurosurgery, and Psychiatry, 91(1), 98–103. 10.1136/jnnp-2019-321354 31527182

[brb32344-bib-0032] Santamaria, J. , Tolosa, E. , & Valles, A. (1986). Parkinson's disease with depression: A possible subgroup of idiopathic parkinsonism. Neurology, 36(8), 1130–1133. 10.1212/WNL.36.8.1130 3736883

[brb32344-bib-0033] Santangelo, G. , Siciliano, M. , Pedone, R. , Vitale, C. , Falco, F. , Bisogno, R. , Siano, P. , Barone, P. , Grossi, D. , Santangelo, F. , & Trojano, L. (2015). Normative data for the montreal cognitive assessment in an Italian population sample. Neurological Sciences: Official Journal of the Italian Neurological Society and of the Italian Society of Clinical Neurophysiology, 36(4), 585–591. 10.1007/s10072-014-1995-y 25380622

[brb32344-bib-0034] Santangelo, G. , Cuoco, S. , Pellecchia, M. T. , Erro, R. , Barone, P. , & Picillo, M. (2018). Comparative cognitive and neuropsychiatric profiles between Parkinson's disease, multiple system atrophy and progressive supranuclear palsy. Journal of Neurology, 265(11), 2602–2613. 10.1007/s00415-018-9038-x 30178175

[brb32344-bib-0035] Schrag, A. , Sheikh, S. , Quinn, N. P. , Lees, A. J. , Selai, C. , Mathias, C. , Litvan, I. , Lang, A. E. , Bower, J. H. , Burn, D. J. , Low, P. , & Jahanshahi, M. (2010). A comparison of depression, anxiety, and health status in patients with progressive supranuclear palsy and multiple system atrophy. Movement Disorders: Official Journal of the Movement Disorder Society, 25(8), 1077–1081. 10.1002/mds.22794 20535826

[brb32344-bib-0036] Siciliano, M. , Santangelo, G. , D'iorio, A. , Basile, G. , Piscopo, F. , Grossi, D. , & Trojano, L. (2016). Rouleau version of the clock drawing test: Age‐ and education‐adjusted normative data from a wide Italian sample. The Clinical Neuropsychologist, 30(sup1), 1501–1516. 10.1080/13854046.2016.1241893 27702066

[brb32344-bib-0037] Streiner, D. L. , & Norman, G. R. , (2008) Health measurement scales: A practical guide to eeir development and use (4th ed.). Oxford University Press.

[brb32344-bib-0038] Uttl, B. , Santacruz, P. , Litvan, I. , & Grafman, J. (1998). Caregiving in progressive supranuclear palsy. Neurology, 51(5), 1303–1309. 10.1212/wnl.51.5.1303 9818850

[brb32344-bib-0039] Visser, M. , Leentjens, A. F. , Marinus, J. , Stiggelbout, A. M. , & van Hilten, J. J. (2006). Reliability and validity of the Beck depression inventory in patients with Parkinson's disease. Movement Disorders: Official Journal of the Movement Disorder Society, 21(5), 668–672. 10.1002/mds.20792 16450355

[brb32344-bib-0040] Wang, Y. P. , & Gorenstein, C. (2012) Psychometric properties of the Beck Depression Inventory‐II: A comprehensive review. Brazilian Journal of Psychiatry, 35(4), 416–431. 10.1590/1516-4446-2012-1048. 24402217

[brb32344-bib-0041] Winter, Y. , Spottke, A. E. , Stamelou, M. , Cabanel, N. , Eggert, K. , Höglinger, G. U. , Sixel‐Doering, F. , Herting, B. , Klockgether, T. , Reichmann, H. , Oertel, W. H. , & Dodel, R. . (2011). Health‐related quality of life in multiple system atrophy and progressive supranuclear palsy. Neuro‐Degenerative Diseases, 8(6), 438–446. 10.1159/000325829 21576919

