# Supplementary figures and images for: Psychometric properties of the Beck Depression Inventory‐II in progressive supranuclear palsy
Source: Brain Behav. 2021 Sep 7;11(10):e2344. doi: 10.1002/brb3.2344 (PMC8553313; doi:10.1002/brb3.2344)

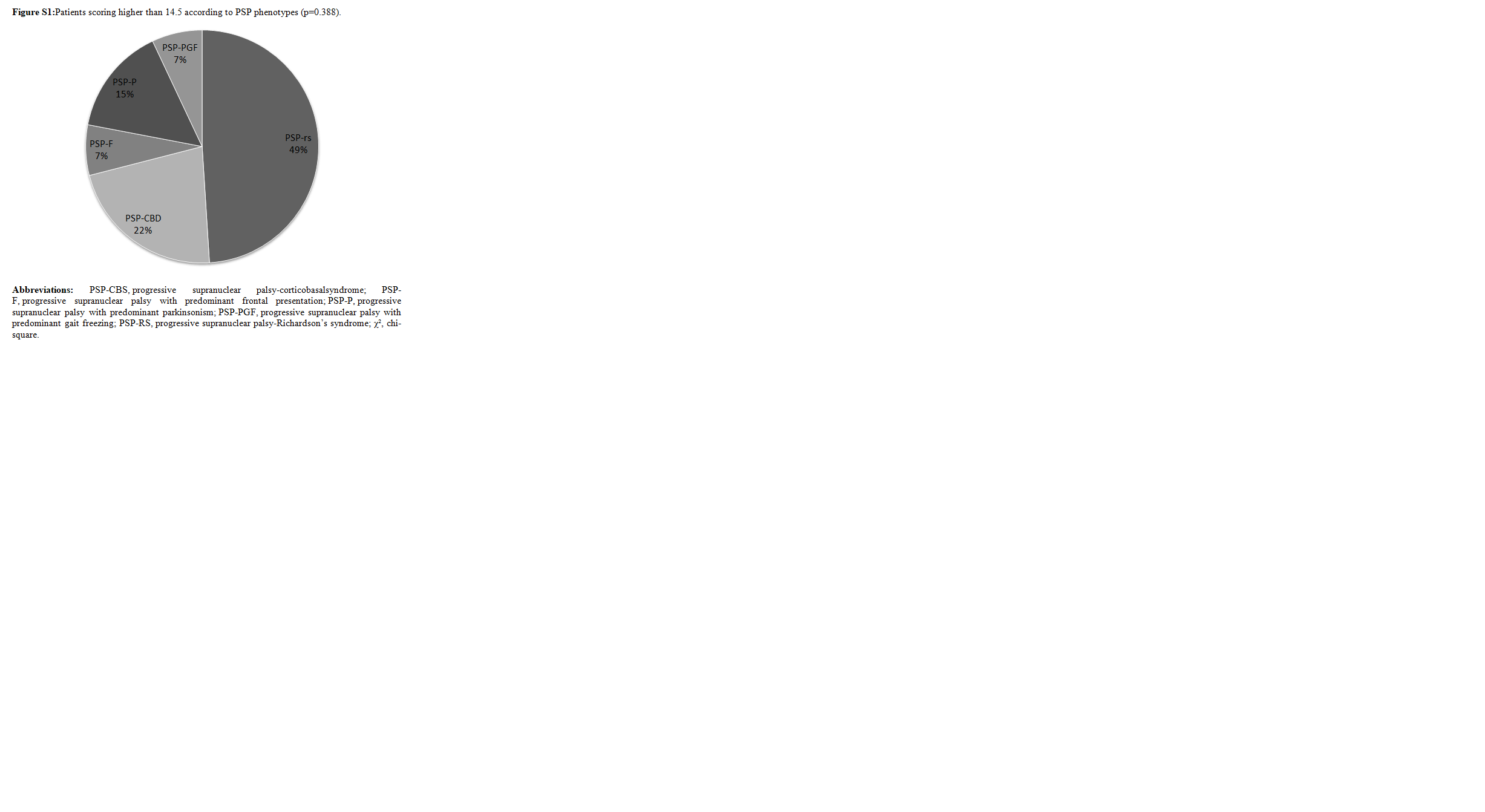

Supplement: Supplementary file 1 — Figure S1 [file BRB3-11-e2344-s002.tif]
