# Supplementary material for: Psychometric properties of the Beck Depression Inventory‐II in progressive supranuclear palsy
Source: Brain Behav. 2021 Sep 7;11(10):e2344. doi: 10.1002/brb3.2344 (PMC8553313; doi:10.1002/brb3.2344)
Supplement: Supplementary file 2 — Table S1 [file BRB3-11-e2344-s003.docx]

**Supplemental Digital Content**

**Table S1:** Correlation between BDI-II items, total score and sub-scores.

|  | **BDI-II**  **Total score**  **(Spearman’s r)** | **BDI-II cognitive sub-score**  **(Spearman’s r)** | **BDI-II somatic-affective sub-score**  **(Spearman’s r)** |
| --- | --- | --- | --- |
| **BDI-II Total score** | - | 0.865^§^ | 0.949^§^ |
| **BDI-II cognitive sub-score** | 0.865^§^ | - | 0.700^§^ |
| **BDI-II somatic-affective sub-score** | 0.949^§^ | 0.700^§^ | - |
| **Item 1** | 0.645^§^ | 0.738^§^ | - |
| **Item 2** | 0.473^§^ | 0.557^§^ | - |
| **Item 3** | 0.138 | 0.184 | - |
| **Item 4** | 0.679^§^ | - | 0.680^§^ |
| **Item 5** | 0.396^§^ | 0.454^§^ | - |
| **Item 6** | 0.294^*^ | 0.491^§^ | - |
| **Item 7** | 0.663^§^ | 0.693^§^ | - |
| **Item 8** | 0.277^*^ | 0.394^§^ | - |
| **Item 9** | 0.547^§^ | 0.538^§^ | - |
| **Item 10** | 0.398^§^ | - | 0.368^§^ |
| **Item 11** | 0.486^§^ | - | 0.506^§^ |
| **Item 12** | 0.625^§^ | - | 0.659^§^ |
| **Item 13** | 0.648^§^ | - | 0.663^§^ |
| **Item 14** | 0.690^§^ | 0.687^§^ | - |
| **Item 15** | 0.684^§^ | - | 0.720^§^ |
| **Item 16** | 0.234 | - | 0.309^*^ |
| **Item 17** | 0.433^§^ | - | 0.362^§^ |
| **Item 18** | 0.498^§^ | - | 0.516^§^ |
| **Item 19** | 0.652^§^ | - | 0.675^§^ |
| **Item 20** | 0.628^§^ | - | 0.629^§^ |
| **Item 21** | 0.354 | - | 0.346^§^ |

*p <0.05

^§^p < 0.01

**Abbreviations:** BDI, Beck Depression Inventory.
