# Supplementary material for: Psychometric properties of the Beck Depression Inventory‐II in progressive supranuclear palsy
Source: Brain Behav. 2021 Sep 7;11(10):e2344. doi: 10.1002/brb3.2344 (PMC8553313; doi:10.1002/brb3.2344)
Supplement: Supplementary file 3 — Table S2 [file BRB3-11-e2344-s001.docx]

**Supplemental Digital Content**

**Table S2:** The frequency of scores 0 (no problem), 1 (mild problem), 2 (moderate problem), 3 (serious problem) of BDI-II items.

| **STATEMENTS** | **0**  **SCORE** | **1**  **SCORE** | **2**  **SCORE** | **3**  **SCORE** |
| --- | --- | --- | --- | --- |
| **Item1:** Sudness | 24/62 (38.7 %) | 25/62 (40.3 %) | 9/62 (14.5 %) | 4/62 (6.5%) |
| **Item 2:** Pessimism | 23/62 (37.1 %) | 26/62 (41.9 %) | 6/62 (9.7 %) | 7/62 (11.3%) |
| **Item 3:** PastFailure | 53/62 (85.5 %) | 7/62 (11.3 %) | 2/62 (3.2 %) | 0/62 (0%) |
| **Item 4:** Loss of Pleasure | 27/62 (43.5 %) | 17/62 (27.4 %) | 10/62 (16.1 %) | 8/62 (12.9%) |
| **Item 5:** Guilty Feelings | 53/62 (85.5 %) | 6/62 (9.7 %) | 2/62 (3.2 %) | 1/62 (1.6%) |
| **Item 6:** Punishment Feelings | 49/62 (79 %) | 3/62 (4.8 %) | 1/62 (1.6 %) | 9/62 (14.5%) |
| **Item 7:** Self-Dislike | 37/62 (59.7 %) | 18/62 (29.0 %) | 4/62 (6.5 %) | 3/62 (4.8 %) |
| **Item 8:** Self-Criticalness | 45/62 (72.6 %) | 14/62 (22.6 %) | 1/62 (1.6%) | 2/62 (3.2%) |
| **Item 9:** SuicidalThoughts or Wishes | 51/62 (82.3%) | 7/62 (11.3 %) | 3/62 (4.8 %) | 1/62 (1.6%) |
| **Item 10:** Crying | 37/62 (59.7 %) | 18/62 (29.0 %) | 4/62 (6.5 %) | 3/62 (4.8%) |
| **Item 11:** Agitation | 27/62 (43.5 %) | 21/62 (33.9 %) | 9/62 (14.5 %) | 5/62 (8.1%) |
| **Item 12:** Loss of Interest | 30/62 (48.4 %) | 17/62 (27.4 %) | 6/62 (9.7 %) | 9/62 (14.5%) |
| **Item 13:** Indecisiveness | 31/62 (50.0 %) | 15/62 (24.2 %) | 7/62 (11.3 %) | 9/62 (14.5%) |
| **Item 14:** Worthlessness | 20/62 (32.3 %) | 23/62 (37.1 %) | 6/62 (9.7 %) | 13/62 (21.0%) |
| **Item 15:** Loss of Energy | 16/62 (25.8 %) | 22/62 (35.5 %) | 14/62 (22.6 %) | 10/62 (16.1%) |
| **Item 16:** Changes in Sleeping Pattern | 23/62 (37.1 %) | 20/62 (32.3 %) | 12/62 (19.4 %) | 7/62 (11.3%) |
| **Item 17:** Irritability | 28/62 (45.2 %) | 24/62 (38.7 %) | 6/62 (9.7 %) | 4/62 (6.5%) |
| **Item 18:** Changes in Appetite | 42/62 (67.7 %) | 6/62 (9.7 %) | 9/62 (14.5 %) | 5/62 (8.1%) |
| **Item 19:** Concentration Difficulty | 30/62 (48.4 %) | 12/62 (19.4 %) | 17/62 (27.4 %) | 3/62 (4.8%) |
| **Item 20:** Tiredness or Fatigue | 20/62 (32.3 %) | 24/62 (38.7 %) | 10/62 (16.1 %) | 8/62 (12.9%) |
| **Item 21:** Loss of Interest in Sex | 41/62 (66.1 %) | 6/62 (9.7 %) | 0/62 (0%) | 15/62 (24.2%) |

**Abbreviation:** 0 Score, absence of symptom; 1 Score, mild symptom; 2 Score; moderate symptom; 3 Score, severe symptom.
